# Concurrent chemoradiotherapy with tomotherapy in locally advanced non-small cell lung cancer: a phase i, docetaxel dose-escalation study, with hypofractionated radiation regimen

**DOI:** 10.1186/1471-2407-13-513

**Published:** 2013-10-31

**Authors:** Alessandra Bearz, Emilio Minatel, Imad Abu Rumeileh, Eugenio Borsatti, Renato Talamini, Giovanni Franchin, Carlo Gobitti, Alessandro Del Conte, Marco Trovò, Tanja Baresic

**Affiliations:** 1 Medical Oncology Department, National Cancer Institute of Aviano (PN), Aviano (PN), Italy; 2 Radiation Oncology Department, National Cancer Institute of Aviano (PN), Aviano (PN), Italy; 3 Nuclear Medicine Department, National Cancer Institute of Aviano (PN), Aviano (PN), Italy; 4 Division of Epidemiology and Biostatistics, National Cancer Institute of Aviano (PN), Aviano (PN), Italy; 5 General Hospital, Pordenone, Italy

## Abstract

**Background:**

Concurrent chemo-radiotherapy is demonstrately superior to sequential chemo-radiotherapy in the treatment of advanced Non-Small-Cell Lung Cancer not suitable for surgery. Docetaxel is considered to enhance the cytotoxic effect of radiotherapy on the tumour cells. Tomotherapy (HT) is a novel radiotherapeutic technique, which allows the delivery of Image Guided-IMRT (IG-IMRT), with a highly conformal radiation dose distribution.

The goal of the study was to estimate tolerability of Docetaxel concurrent with IMRT and to find the maximum tolerated dose of weekly Docetaxel concurrent with IMRT delivered with HT Tomotherapy after induction chemotherapy with Cisplatin and Docetaxel in patients affected with stage III Non-Small Cell Lung Cancer.

**Methods:**

We designed a phase I, dose-finding study to determine the dose of weekly Docetaxel concurrent with Tomotherapy after induction chemotherapy, in patients affected by Non-Small Cell Lung Cancer with Stage III disease, not suitable for surgery.

**Results:**

Concurrent weekly Docetaxel and Tomotherapy are feasible; we did not reach a maximum tolerated dose, because no life-threatening toxicity was observed, stopping the accrual at a level of weekly docetaxel 38 mg/m^2^, a greater dose than in previous assessments, from both phase-I studies with weekly docetaxel alone and with Docetaxel concomitant with standard radiotherapy.

**Conclusions:**

Concurrent weekly Docetaxel and Tomotherapy are feasible, and even with Docetaxel at 38 mg/m^2^/week we did not observe any limiting toxicity. For those patients who completed the combined chemo-radio treatment, median progression-free survival (PFS) was 20 months and median overall survival (OS) was 24 months.

## Background

Concurrent chemo-radiotherapy is considered the standard treatment for patients affected by locally advanced, stage III, unresectable Non-Small Cell Lung Cancer (NSCLC) and good performance status (PS) [1]. Stage III NSCLC constitutes a heterogeneous group, likely due to a different nodal involvement; its median survival has been recently updated from 12 to 23.3 months in phase III trials [2,3]. The recommended systemic treatment in stage III NSCLC consists of 2–4 cycles of platin-combination with concurrent radiotherapy [4]. In an effort to improve overall survival (OS) different schemes for both induction and consolidation therapy are used in clinical practice, with induction chemotherapy before the start of concurrent chemo-radiotherapy preferred by some [2,5] and consolidation modalities preferred by others [3,6]. However, no clear superiority has been demonstrated between those two approaches. Docetaxel enhances the cytotoxic effects of radiotherapy in vitro [7]. In several phase II studies radiotherapy with concurrent Docetaxel and after induction with Cisplatin-Docetaxel combinations has been shown to be feasible [8,9], with encouraging OS rates. There is an indication that higher radiation doses may result in improved probability of local tumour control [10]; however, using current radiation delivery techniques dose escalation, in particular in combination with chemotherapy, may lead to unacceptable lung toxicity. Therefore, the search for better radiotherapy approaches is important and Tomotherapy (HT) may be one of the most promising technologies for this purpose [11]. HT is a novel technique, which allows the delivering of Image Guided - IMRT (IG-IMRT), by using a dynamic delivery in which gantry, treatment couch, and multileaf collimator leaves are all in motion during treatment, resulting in a highly conformal radiation dose distribution [12]. The development of those techniques has led to improved radiation delivery with better tumor coverage and decreased exposure of surrounding normal tissues; however, in the CALBG 30105 study [13] the arm with Gemcitabine concurrent with high dose conformal radiation treatment was closed for high rate of severe pulmonary toxicities, likely due to larger mean lung doses; however the authors suggest that radiosensitizing properties of Gemcitabine on normal tissue may have contributed to the higher pulmonary toxicity observed [14]. On this basis, we were concerned that, using a radiosensitizer agent such as Docetaxel concurrent with Tomotherapy, with larger volumes of healthy lung treated with low dose radiation, could lead to a different maximum tolerated dose (MTD) for Docetaxel.

We designed a phase I, dose-finding study to estimate the tolerability of concurrent Docetaxel with Tomotherapy and to determine the MTD of weekly Docetaxel concurrent with IMRT delivered with HT after induction chemotherapy. We chose to study Docetaxel alone combined with concurrent HT, because Docetaxel may enhance both activity and toxicity of radiotherapy and we did not want to have any confounding bias in the results analysis. For this reason we chose to deliver the backbone of systemic treatment for NSCLC, i.e. platinum, in the induction part, using Docetaxel alone in the concurrent study.

## Methods

This was a mono-institutional, phase I, dose-finding study. Enrolment started in March 2008 and was closed in June 2011. Eligible patients were male or non-pregnant and non-breast feeding females, aged more than 18 years, PS ECOG 0 or 1, with histologically or cytologically confirmed NSCLC with multiple clinical-level N2 or N3 for mediastinal lymph nodes, above the 15 mm short-axis threshold at Computed Tomography (CT) scanning. Patients with supraclavicular involvement of lymph nodes and pleural effusion were excluded. Fluoro-2-deoxy-D-glucose-positron emission tomography (FDG-PET) fusions with CT (PET-CT), as well as CT-scans of the thorax, blood tests and pulmonary function tests were mandatory at baseline. Patients must have adequate organ function including the following: bone marrow reserve (white blood cell count ≥3.0 × 10^9^/L, absolute neutrophils count ≥1.5 × 10^9^/L), hepatic function (bilirubin ≤1.5 times upper limits of normal [x ULN] and alkaline phosphatase, aspartate transaminase and alanine transaminase ≤2.5 × ULN), and renal function (serum creatinine ≤1.5 × ULN). Screening assessments were carried out within 28 days of the first dose of chemotherapy. The treatment protocol was reviewed and approved by the competent authorities and the institutional ethics committee and was registered with the authorities (European Union Drug Regulating Authorities Clinical Trial no. EUDRACT 2008-001074-33). Each patient signed an informed consent document.

### Treatment plan

In the induction part all patients received three cycles of Cisplatin 80 mg/ m^2^ and Docetaxel 75 mg/m^2^ on day 1, every three weeks. A PET-CT scan was repeated after the third cycle and patients with stable disease or partial/complete response were included in the phase I radiotherapy concurrent with chemotherapy protocol. The phase I, dose-escalation trial consisted of 5 weeks of once-weekly Docetaxel (on day 3 of every week) concurrent with radiotherapy at a dose of 2,4 Gy/day for 5 days/week for 5 consecutive weeks to a total dose of 60 Gy. Docetaxel dosage was scheduled as 10 mg/m^2^ weekly for the first 3 patients; if no severe toxicity occurred, the next 3 patients were to be treated with Docetaxel 13 mg/m^2^/week, and so on according to a modified Fibonacci 3 + 3 design [15]. The dose was escalated in cohorts of 3 patients and 3 mg/m^2^ were added to every cohort. The choice to add 3 mg/m^2^ at each cohort instead of the 67% of the starting dose like in Fibonacci design was due to the absence of information about HT concurrent to chemotherapy. At the time this protocol was planned, it was only known the dose limitation of 30 mg/m^2^/week for Docetaxel concomitant with radiotherapy [16]; for this reason the dose was increased with caution. Dose-limiting toxicities were defined as grade (G) 4 thrombocytopenia, or at least G2 bleeding, G4 neutropenia, or febrile neutropenia, G3 nausea, vomiting or diarrhoea; unexpected G2 toxic effects needing dose reduction or delay in the concurrent treatment were also classified as dose limiting. In the case of at least one severe toxicity, 3 more patients had to be added at that level and, if again severe toxicity occurred within the same cohort, that dose was to be considered the maximum tolerated dose, with the previous level chosen as the dose to use in concurrent modality with HT. Adverse events were graded according to National Cancer Institute Common Terminology Criteria for adverse events (version 3.0) [17].

### Chemotherapy

Docetaxel was administered in a 60-minute i.v. infusion, both in induction (75 mg/m^2^ every three weeks) and concurrent therapy (weekly and with dose-escalation). The standard prophylaxis to prevent hypersensitivity reactions included a single intravenous administration of corticosteroids and histamine-blocking drugs just before Docetaxel infusion. Induction Cisplatin 80 mg/m^2^ was administered as a 60-minutes i.v. infusion immediately following the Docetaxel infusion. Hydration and prophylactic antiemetics were administered before chemotherapy. In the induction part dose modifications allowed due to toxicity were: Docetaxel 75% for febrile neutropenia, G4 neutropenia, febrile neutropenia, G4 thrombocytopenia, G ≥3 mucositis; and Cisplatin 75% for nephrotoxicity G ≥ 2.

### Concurrent chemo-radiotherapy

No dose modifications for Docetaxel in concurrent modality were allowed. Docetaxel was administered on day 3 every week of radiotherapy, and thoracic radiotherapy was started 30 minutes after infusion.

### Radiotherapy

Patients underwent a volumetric treatment-planning CT scan using an individualized immobilization device in the treatment position on a flat table. The gross tumour volume (GTV), planning target volume (PTV) and planning organ at risk volume (PRV) were delineated according to the International Commission on Radiation Units and Measurements (ICRU) Report 62 guidelines. The restaging FDG-PET/CT performed before radiation therapy was co-registered to the treatment-planning CT to improve the target delineation. The GTV included the primary tumour and any FDG-avid regional lymph nodes. For this study, the clinical target volume was equivalent to the GTV. Elective nodal regions were not intentionally irradiated. The PTV volume included the GTV with a minimal 3D margin of 10 mm. A 4D CT-scan was used to check GTV and PTV margins with respect to physiologic ventilatory tumour excursion. The dose prescribed to the PTV was 60 Gy delivered in 25 fractions (2.4 Gy/fraction), so that 95% of the PTV received the 98% of the prescribed dose. Using the linear quadratic model and the BED equation derived from this model, assuming an α/β ratio of 10 Gy, this prescription would be equivalent to 66 Gy in a standard 2-Gy fractionation [18,19]. There are two modalities of shortening overall time of radiotherapy without increasing late complications and allowing for better efficacy outcomes: first is two fractions per day, second is using fewer and larger doses [20,21]. We used a relatively hypofractionated radiation regimen based on the second modality. Specific dosimetric guidelines were the following: spinal cord maximum dose <46 Gy; mean lung dose <14 Gy; lung V20 (percentage of lung receiving 20 Gy) <25%. Treatment was delivered once a day, five fractions weekly. All patients were treated with HT [11]. A Megavolt CT-scan was also performed daily for each patient to image-guide the radiation treatment, no adaptive radiotherapy was performed. Patients were seen weekly during the radiotherapy course to determine the presence of symptoms.

### Patient evaluation

All patients underwent a full physical examination (including determination of performance status, weight loss, vital signs), and haematological test at baseline and then weekly during induction and concurrent therapy. They also underwent a biochemistry test at baseline and every 3 weeks during induction and concurrent treatment. Chest CT was mandatory and brain CT was performed if clinically indicated; all patients had multi-level N2 or N3 involvement demonstrated both by CT scan and PET-CT. Tissue or cytologic diagnosis was made using biopsy/brushing or bronchial aspirate obtained during fibre-optic bronchoscopy or, alternatively, transthoracic aspiration biopsy of the primary tumour. CT-PET scan was performed ≤2 weeks before starting induction chemotherapy and local chemo-radiotherapy, respectively, and was repeated every 4 months during follow-up, starting two months after the end of concomitant treatment. PET/CT scans were performed using a combined PET and CT system (Discovery LS PET CT, GE Healthcare, Milwaukee, WI, USA). Patients were fasting for at least 6 hours before tracer injection and each patient received 350–400 MBq of FDG (^18^ F-Fluoro-2-deoxy-D-glucose) intravenously. PET/CT acquisitions were carried out at 60 minutes post tracer-injection, after the patients waiting for the scan were kept at rest in a quiet, dimly lit room. A whole-body PET/CT scan, consisting of helical CT scanning from the head through the pelvis (rotation time 0.8 s, slice thickness 5 mm, image interval 4.25 mm, X-ray voltage 140 kV and X-ray current 80–140 mA), followed by a 2D PET acquisition (six or seven bed positions at 5 min per bed position) was performed in all patients. Immediately after being acquired, images were reconstructed using ordered subsets expectation maximization 2D iterative reconstruction; CT images were used to produce attenuation correction values for PET emission reconstruction and fused PET/CT presentation, in order to avoid misinterpretation between inflammatory pathology and tumour. PET data were interpreted independently of the result of any prior investigation by two nuclear medicine physicians in consensus, expert in oncological imaging. Any focal FDG uptake in a pulmonary lesion or in a mediastinal node higher than the surrounding background activity was used as a PET criterion for malignancy. In particular, high focal lung tracer uptake was interpreted as cancer uptake in disease staging and as persistent disease in re-staging. Focal activity in mediastinal lymph nodes was considered as metastatic disease when consistent with an abnormal volumetric size by CT-scan. Assessment of FDG uptake was made visually, comparing PET/CT studies of the same patient, considering the visual increasing or decreasing FDG uptake in the lesions together with RECIST morphological criteria of response. The standardized uptake value of FDG uptake (SUV) was used as an accessory reference only. The results of PET-CT were then classified as progressive disease, stable disease, or partial and complete remission in according to both PET visual FDG uptake analysis and CT morphological criteria.

The primary endpoint was safety and toxicity of Docetaxel concurrent with Radiation Therapy after induction treatment in order to define the maximum dose level. Any treatment-related side effects were followed until resolution. Tumour response was assessed according to RECIST criteria 3.0 [22].

The efficacy of treatments was evaluated in all patients who were allocated to induction treatment; response rate was recorded and follow-up for progression free survival (PFS) and overall survival (OS) was maintained till progression and death for all patients who completed the induction and concurrent treatment. Kaplan-Meier methods were used to assess PFS and OS [23].

## Results

The first patient was enrolled in January 2008, the last one in January 2011. Analysis was done in June 2012; a total of 37 patients were treated. Four patients (10%) out of 37 did not go into the concurrent chemo-radiotherapy, 2 because of progression of disease after the induction therapy and 2 because of consent withdrawal. Patients’ characteristics are summarized in Table 1. Seven patients were female (19%) and 30 males (81%); median age was 61 years (range 40–77), 16 patients were affected by squamous cell carcinoma (43%) and 20 by adenocarcinoma (54%); one patient (3%) had an undifferentiated histology. For 23 patients only EGFR mutational analysis was possible, and only one patient (4%) harboured a mutation at exon 21. About Performance Status according to ECOG scale at diagnosis, 22 patients (60%) had PS 0, and 15 (40%) had PS 1. Fifteen patients (40%) had stage IIIA and 22 (60%) IIIB disease. Docetaxel was reduced at 75% of the planned dose for toxicity in 4 patients (11%) during the induction treatment: 2 patients showed mucositis G3, one patient had nausea G3 and one patient suffered from atrial fibrillation. During induction therapy, 5 patients (13%) reported neutropenia G3, and 3 (8%) neutropenia G4 without fever. G-CSF was used as primary prophylaxis in 11 patients (30%), from cycle II in the induction treatment. No thrombocytopenia or anaemia greater than G1 was recorded. Three patients had nausea G3 and one patient reported diarrhoea G3 in the induction phase. All 37 patients were evaluable for response to induction chemotherapy: the ORR was 31/37 (84% all partial responses), 2 progressive diseases (5%), and 4/37 patients were stable (10%). Thirty-three patients were subsequently enrolled into the phase I dose escalation study, with concurrent chemo-radiotherapy. All 33 patients received the planned radiotherapy total dose of 60 Gy and weekly Docetaxel; each cohort received 3 mg/m^2^ more of weekly docetaxel, starting from 10 mg/m^2^ to 38 mg/m^2^ (Table 2). No life-threatening haematological toxicities were recorded in the concurrent treatment, 2 patients had leucopoenia G2; radiation pneumonitis were never reported; no patient reported diarrhoea; esophagitis was G2 in 7/33 patients (21%), and G3 in one patient (3%). No late oesophageal toxicity was observed. Docetaxel dose-escalation followed the standard 3 + 3 rule, starting from 10 mg/m^2^/week. In the absence of dose-limiting toxicity (DLT), we decided to stop at a dose of 38 mg/m^2^/week, a greater dose than previous assessments in phase-I studies with weekly docetaxel alone [24].

**Table 1:** Demographics of 37 patients

		**Patients (%)**
Median age (years)	61 (44–77)	
Sex		
Females		7 (19)
Males		30 (81)
Histology		
Squamous		16 (43)
Adenocarcinoma		20 (54)
Undifferentiated (NOS)		1 (3)
PS ECOG		
0		22 (60)
1		15 (40)
Stage		
IIIA		15 (40)
IIIB		22 (60)

**Table 2:** **Dose**-**level evaluation of 33 patients**

**Dose level**	**Weekly docetaxel** (**mg**/**m**^**2**^/**week**)	**N**. **of patients with DLT/treated patients**	**DLT**
1	10	0/3	
2	13	1/6	Grade 3 esofagitis
3	16	0/3	
4	19	0/3	
5	22	0/3	
6	25	0/3	
7	28	0/3	
8	31	0/3	
9	35	0/3	
10	38	0/3	

**Figure 1:**
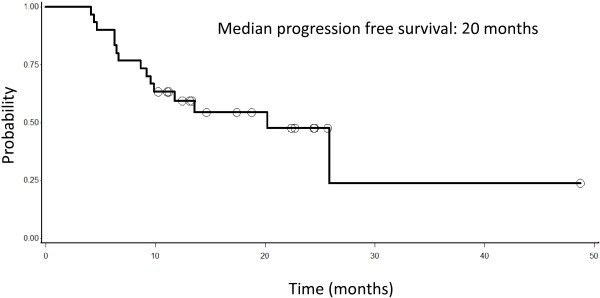
**Progression free survival of 33 patients with lung cancer**, **stage III**, **not operable and treated with induction chemotherapy and radiotherapy.**

**Figure 2:**
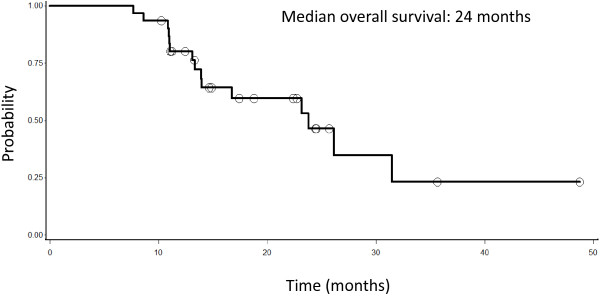
**Overall survival of 33 patients with lung cancer**, **stage III**, **not operable and treated with induction chemotherapy and radiotherapy.**

Response at the first PET-CT scan after the end of concurrent chemo-radiotherapy was as follows: 28 patients (85%) showed a partial response, 2 patients (6%) had a progression of disease, 2 patients were stable (6%). Median PFS was calculated for 33 patients who completed the combined chemo-radio treatment, and it was 20 months (Figure 1). Median OS for the 33 patients who received both induction chemotherapy and concomitant chemo-radiotherapy was 24 months (Figure 2).

## Discussion

The treatment of locally advanced NSCLC has been a controversial issue. It has been well established that concurrent chemo-radiotherapy with Etoposide and Cisplatin improves survival compared with radiation alone [1] and it is considered the standard of treatment for locally advanced NSCLC. Many other strategies have been tried in order to overcome the achieved survival plateau, established around a median 17 months [1]. To date, no randomized comparisons of consolidation chemotherapy versus observation after concurrent chemo-radiation have shown a survival benefit in locally advanced NSCLC [3,25]. The induction chemotherapy strategy has several theoretical advantages, such as increasing the sensitivity of tumours in early-stage disease, decreasing the tumour volume to enable a better local control by radiation, allowing faster eradication of micro-metastatic disease. Several randomized phase II studies were conducted in order to explore the efficacy of induction chemotherapy followed by concurrent chemo-radiation versus concomitant treatment followed by consolidation chemotherapy; however no statistically significant differences were observed [2,26]. The introduction of taxanes, Gemcitabine and Vinorelbine in combinations with platin compounds as well as the introduction of new techniques of radiotherapy may improve the therapeutic efficacy of chemo-radiation combination; nevertheless, the optimal combination, dose intensity, strategy or timing is still to be defined. In order to study the feasibility of concurrent chemotherapy with HT, a highly conformal, new radiation technique, we decided to associate Docetaxel, known as radio-sensitizing chemotherapeutic agent. Our goal was to perform a dose-finding study to individuate the maximum tolerated (MTD) dose of Docetaxel when associated with HT, assuming that MTD could be different from the one already known of Docetaxel concurrent with standard radiation therapy. In order to avoid confounding bias, we decided not to associate Docetaxel with Cisplatin during the concomitant treatment. Being that it is not ethical to treat patients without Cisplatin, we decided to offer them an induction treatment with three cycles of Cisplatin and Docetaxel before concurrent chemo-radiation, although a previous phase III study by Vokes EE et al. had already stated that induction chemotherapy does not add a significant survival benefit over concurrent therapy alone [2]. For this reason, we decided that patients after receiving three cycles of induction with Cisplatin and Docetaxel were treated with radiation therapy concurrent with weekly Docetaxel, within a dose-finding study for the weekly Docetaxel. All the patients had multi-level N2 or N3 lymph nodal involvement, confirmed mostly by a non invasive modality, both CT-scan and PET-CT. In a recent meta-analysis, PET scanning was superior to CT scanning in the detection of nodal metastatic disease, with sensitivity of 83% and specificity of 92% [27]. In an attempt to determine the need for invasive staging like mediastinoscopy after PET and CT imaging, a meta-analysis evaluated the association between the size of mediastinal lymph nodes and the probability of malignancy [28]. The authors concluded that the prevalence of metastasis strongly increases above the 15 mm short-axis threshold at CT scanning, positioning the need for mediastinoscopy only in patients with nodes measuring <15 mm on CT and negative on FDG-PET or with hilar involvement of the tumour. We decided to include only patients with nodes > 15 mm short-axis at CT-scanning and positive on FDG-PET, and for this reason we do not performed mediastinoscopy at staging.

We did not reach a maximum tolerated dose, because no life-threatening toxicity was observed. We decided to stop the accrual early at a level of weekly docetaxel 38 mg/m^2^, that being a greater dose than in previous assessments, from both phase-I studies with weekly Docetaxel alone and with Docetaxel concomitant with standard radiotherapy. To our knowledge this is the first study of Tomotherapy with weekly concurrent chemotherapy in a dose escalation study of Docetaxel, in the treatment of NSCLC. There was a previous published study of Tomotherapy and concurrent chemotherapy, with fixed dose of chemotherapy, Cisplatin and Docetaxel at a dose of 20 mg/m^2^/weekly, each administered weekly and with radiotherapy fraction size escalation [29]. Although it was not a primary endopoint for this study, the PFS and OS of the patients recruited in the study are encouraging, well above the median data obtained in the previous studies with chemo-radiation for locally advanced NSCLC; however they need further validation through phase II, multi-institutional studies and eventually comparative phase III trials.

## Conclusion

Tomotherapy and concurrent chemotherapy with Docetaxel is feasible in advanced NSCLC patients and warrants further multi-institutional validation trials.

## Competing interests

The authors declare that they have no competing interests.

## Authors’ contributions

AB, EM, GF, AIR, CG, ADC, MT carried out the clinical work, EB and TB performed the imaging analyses, RT carried out the statistical analyses, AB drafted the manuscript. All authors read and approved the final manuscript.

## Pre-publication history

The pre-publication history for this paper can be accessed here:

http://www.biomedcentral.com/1471-2407/13/513/prepub
